# Digital health: current applications, challenges, and future directions for enhancing healthcare quality and safety

**DOI:** 10.3389/fpubh.2025.1646802

**Published:** 2025-09-26

**Authors:** Saidi Hu, Danyang Song, Siran Wan, Shunhong Zhang, Chenchen Luo, Nian Li, Guangyue Liu, Jailson da Graça Espírito Santo Vasconcelos, Leonilde Lavres Ceita de Carvalho, Eveline Neobísi, Monazeri Lima Bragança da Costa, José Etchu Takounjou, Karem Maimite Das Neves, Luzimery dos Ramos da Conceição, Marinela da Costa Encarnação, Lin-Yong Zhao

**Affiliations:** ^1^Department of Stomatology, Yaan People’s Hospital, Yaan, China; ^2^Department of Gynaecology and Obstetrics, Yaan People’s Hospital, Yaan, China; ^3^Department of Cardiology, Pangang Group General Hospital, Panzhihua, China; ^4^Department of Outpatient Chengbei, The Affiliated Stomatological Hospital, Southwest Medical University, Luzhou, China; ^5^Department of Traditional Chinese Medicine, Panzhihua Central Hospital, Panzhihua, China; ^6^Department of Anesthesiology, West China Hospital, Sichuan University, Chengdu, China; ^7^Department of General Surgery, The Democratic Republic of São Tomé e Príncipe, Hospital Dr Ayres de Menezes, Sao Tome, Sao Tome and Principe; ^8^Delegação de saúde São Tomé e Príncipe, The Democratic Republic of São Tomé e Príncipe, Sao Tome, Sao Tome and Principe; ^9^The Democratic Republic of São Tomé e Príncipe, Hospital Dr Ayres de Menezes, Sao Tome, Sao Tome and Principe; ^10^Department of General Surgery and Laboratory of Gastric Cancer, State Key Laboratory of Biotherapy/Collaborative Innovation Center of Biotherapy and Cancer Center, West China Hospital, Sichuan University, Chengdu, China; ^11^Gastric Cancer Center, West China Hospital, Sichuan University, Chengdu, China

**Keywords:** digital health technologies, healthcare quality and safety, COVID-19, healthcare access disruption, digital divide, wearable devices, artificial intelligence

## Abstract

Digital Health Technologies (DHTs) have become a cornerstone of modern healthcare, significantly improving quality and safety across clinical practice, public health, and medical research. Originating in the mid-to-late 20th century, DHTs have facilitated substantial progress in personalized medicine, predictive analytics, and remote patient monitoring through the implementation of artificial intelligence (AI), wearable devices, and telemedicine platforms. During the Coronavirus Disease 2019 (COVID-19) pandemic, these technologies proved indispensable for epidemic surveillance and precision containment, while also mitigating healthcare access disruptions. Nevertheless, critical challenges including the digital ethics and equity, technical and regulatory policy restrictions, privacy and data security concerns, and clinical workflow integration issues remain to be addressed. This narrative review explores the transformative role of DHTs throughout the disease management continuum—from prevention to prognosis—and evaluates their contributions to healthcare quality and safety. It also provides strategies for stakeholders to address existing barriers. By overcoming these challenges, DHTs can further elevate healthcare standards, fostering a safer and more efficient global healthcare system.

## 1 Introduction

Driven by the information revolution, digital health has emerged as a transformative force in healthcare, with its framework and applications continuously evolving. According to the World Health Organization (WHO), digital health refers to the use of technology, particularly internet-based tools, for diagnosing, monitoring, treating, and preventing diseases (1). It also encompasses the interdisciplinary field dedicated to the strategic application of technology to enhance both individual and population health, as well as improve patient care (2). Digital health plays an increasingly vital role across various sectors of healthcare. The Coronavirus Disease 2019 (COVID-19) pandemic underscored the critical role of Digital Health Technologies (DHTs), such as telemedicine and the Internet of Things (IoT) (3) in mitigating healthcare disruptions and strengthening epidemic surveillance (4). These technologies have not only improved access to medical services but also ensured the swift delivery of high-quality health information, a cornerstone of clinical decision-making and healthcare safety (5).

Traditional healthcare systems face persistent challenges, including diagnostic errors, inefficiencies, and disparities in resource allocation (6). DHTs, such as Electronic Health Records (EHRs) (7) and Patient-Generated Health Data (PGHD) (1), address these issues by centralizing medical information and empowering patients to participate in their care. Digital devices are driving transformative change, enhancing clinicians’ ability to diagnose and treat patients accurately, and enabling more informed decision-making (8). Beyond traditional care, DHTs—particularly digital therapeutics (DTx)—address key limitations in clinical practice by reducing regional disparities in access, lowering costs, enhancing population health, and promoting socioeconomic stability (9, 10). Remote patient monitoring (RPM) systems enable clinical teams to continuously collect physiological parameters from patients with chronic diseases—such as heart failure (HF), diabetes—and provide early warnings and interventions. These systems have been shown to reduce first heart failure readmissions by up to 22% (11) and cardiovascular mortality by 3.46% (12). To realize these benefits, DHT implementations should prioritize user-centered design and leverage interoperable technologies—including artificial intelligence (AI), sensor networks, and 3D printing—to enhance healthcare efficiency and accessibility (13). Such integrated systems must enable seamless data exchange and transform medical information into actionable clinical insights, while addressing existing implementation barriers in digital healthcare (14). The WHO’s Global Strategy for Digital Health 2020–2025 emphasizes the need for ethical, equitable, and sustainable integration of these technologies into health systems (15).

This narrative review examines the applications of digital health across prevention, diagnosis, treatment, prognosis, public health, education, and research, with a focus on improving healthcare quality and safety. It provides insights into the digital ethics and equity, technical and policy restriction, privacy and data security concerns, and workflow integration, offering valuable guidance to policymakers and practitioners navigating the evolving healthcare landscape.

## 2 Methodology for literature selection

### 2.1 Literature search strategy

This narrative review synthesizes the current landscape, applications, and challenges of DHTs with a specific focus on healthcare quality and safety. To identify relevant literature, a comprehensive search was conducted across electronic databases including PubMed/MEDLINE, IEEE Xplore, and Google Scholar for articles published up to July 2025. Search terms encompassed a combination of keywords related to digital health. The search strategy included a combination of terms and free-text keywords related to digital health and healthcare outcomes. The search strategy was as follows: (“digital health” OR “digital medicine” OR “e-health” OR “m-health” OR “telemedicine” OR “wearable devices” OR “artificial intelligence” OR “AI” OR “machine learning” OR “deep learning” OR “digital therapeutics” OR “remote patient monitoring” OR “EHR” OR “electronic health records”) AND (“healthcare quality” OR “patient safety” OR “clinical outcomes” OR “diagnostic accuracy” OR “treatment efficacy” OR “health equity” OR “access to care” OR “health disparities” OR “prevention” OR “diagnosis” OR “treatment” OR “prognosis” OR “public health” OR “medical education” OR “privacy” OR “data security” OR “interoperability” OR “digital divide” OR “regulatory policy”). Both primary research studies and high-impact reviews were considered.

### 2.2 Inclusion and exclusion criteria

The inclusion criteria prioritized: (1) studies focusing on the application of DHTs in clinical or public health settings; (2) articles reporting on measurable impacts on quality, safety, or efficiency; (3) discussions of implementation challenges and solutions.

Exclusion criteria included: (1) articles not available in English; (2) studies solely focused on technical engineering details without clinical correlation; (3) opinion pieces without empirical evidence.

Given the narrative nature of this review, a formal quality assessment or risk-of-bias analysis was not performed. However, emphasis was placed on citing evidence from peer-reviewed journals, major conference proceedings, and reports and policy documents from authoritative organizations like the World Health Organization (WHO).

### 2.3 Search results

During the preparation of this review, we referenced a systematic literature screening process (see Supplementary Figure 1) based on PRISMA guidelines. This process initially identified a substantial number of records from multiple databases. After deduplication, automated screening, and manual evaluation, 128 studies and 10 reports (including reports, major conference proceedings and policy documents) were ultimately included as part of the evidence foundation. Although this review is not a systematic evaluation, the screening process helped outline the current research landscape in digital health and supported the presentation of existing evidence in this paper.

## 3 The origin and development of digital health

The concept of digital health emerged in the mid-to-late 20th century with the initial integration of computer technology into healthcare, notably through the development of EHRs and early telemedicine initiatives (16). For example, the first use of two-way interactive closed-circuit television for psychiatric patient consultations at the University of Nebraska in 1959 is regarded as the starting point of modern telemedicine (17). In 1968, Dr. Lawrence Weed’s proposal of the Problem-Oriented Medical Record (POMR) became a precursor to modern structured EHRs (18), and early EHR systems such as COSTAR (19) and HELP were among the first outpatient electronic medical record systems. In the early 21st century, the proliferation of wearable technology and mobile internet fueled its expansion into telemedicine, health monitoring, and big data analytics (20). Advancements in blockchain (21) and AI after 2010 further propelled the growth of precision medicine and personalized health management. The rapid rise of the internet has fostered the development of a digital health ecosystem, encompassing wearable devices, electronic health (e-health), mobile health (m-health), medical imaging, telemedicine, genomics, and information systems, among others (22).

The COVID-19 pandemic significantly accelerated the adoption of DHTs, resulting in widespread use of online consultations and health data tracking. As a direct consequence, digital health platforms and technologies have played a key role in gathering and analyzing diverse health data, aiding in understanding the virus’s behavior, and facilitating the development of effective treatments and vaccines, thereby reducing transmission and saving lives (23). However, some scholars argue that the COVID-19 pandemic itself is a product of digitalization and technological progress (24).

As digitalization becomes an essential trend for the survival of healthcare organizations (25), numerous countries have implemented policies and regulations concerning digital healthcare. The Riyadh Declaration highlights the importance of data, digital technologies, and innovation in building resilient healthcare systems (26). Additionally, the WHO has launched the Global Initiative for Digital Health (GIDH) to support digital health transformation globally (27). As DTx evolve, they hold significant potential to transform healthcare by enhancing and complementing traditional therapies (28). Among diabetic patients receiving digital cognitive behavioral therapy (CBT), medication adherence improved, and HbA1c levels decreased by 0.39% compared to usual care over a 3-month observation period (29).

## 4 Digital health offers a broad spectrum of applications in healthcare

### 4.1 Clinical prevention

Digital health has given rise to numerous clinical prevention technologies, including wearable devices, AI, digital twins, remote monitoring, and ultrasound-based virtual modeling. Wearable technologies, such as smartwatches and adhesive patches, have gained significant popularity for health monitoring. By 2024, the global user base is expected to reach 224.31 million, with 92% using these devices for health and fitness purposes (30). These devices track physical activity, sleep patterns, and physiological data, such as heart rate and blood sugar levels, facilitating early disease prevention (31). Although approximately 60% of epileptic seizures remain undetected by patients or healthcare providers (32), wearable devices can identify seizures and issue real-time alerts, enhancing safety during and after an event (33). For atrial fibrillation (AF) management, wearable devices enable continuous heart rate monitoring and real-time detection of arrhythmia onset (34). When integrated with AI and e-health devices, these technologies can efficiently process vast amounts of medical data, revealing underlying patterns and predicting disease progression. Such applications not only reduce the risk of stroke in individuals with AF but also play a pivotal role in preventing and managing various chronic conditions (35).

Digital twins are virtual replicas of human cells, tissues, organs or microenvironments that can continuously adapt to real-time changes in results (36). The combination of wearable devices and mobile applications with twin technology offers personalized predictive analytics through real-time health data monitoring, augmented by virtual simulations, to detect early anomalies (37). By integrating digital twin technology with wearable devices and mobile apps, individuals can manage their health more effectively, tracking physical activity, eating habits, and sleep patterns, thus gaining insights to make healthier lifestyle choices. The data collected is not only valuable for users but also for healthcare providers, who can leverage it to create personalized disease prevention programs tailored to specific patient needs.

Through advanced modeling and digital twins, DHTs are transforming reproductive health management by improving genetic and prenatal assessments, enhancing reproductive health, and reducing future healthcare burdens. For example, by analyzing vaginal microbiome data, the Deep Learning Model for Preterm Birth Prediction (DeepMPTB) predicted preterm birth risk with 84.10% accuracy, providing clinicians a valuable tool for improved risk assessment and personalized intervention (38). Similarly, digital twin technology constructs precise virtual pregnancy models for clinical applications, enabling healthcare providers to evaluate tissue integrity in cases of scarred uterine pregnancies, assess the risks of uterine rupture, and implement preventive measures against pregnancy complications (39). These complementary digital methods collectively enhance prenatal care and risk management.

To unlock the full potential of clinical prevention, DHTs must be applied comprehensively. For instance, in diabetes management, AI analyzes group data to identify risk factors such as hypertension, smoking, physical inactivity, and poor diet, promoting “AI-driven personalized mobile medicine” to prevent the disease (40). Diabetic patients require lifelong personalized management with continuous glucose monitoring (CGM) being especially critical (41). The CGM devices using subcutaneous sensors to measure blood glucose levels (42), while the system integrates remote monitoring and AI to share in real-time data and predict blood sugar fluctuations (43), alerting patients before complications arise and facilitating early medical intervention (44).

### 4.2 Clinical diagnosis

For diagnosis, clinicians mostly rely on quick and precise picture interpretation. While DHTs like AI, EHR, and imaging technology are combined, diagnostic accuracy and efficiency can be substantially improved. Complex AI systems are particularly excellent at diagnosing problems (8). Complex data sets can be analyzed by AI systems, which can then spot abnormal patterns that might indicate cancer (45). They excel in radiological diagnostics, have greater capabilities to detect subtle abnormalities that human doctors may miss, and diagnose arthritis or severe injuries (46). Artificial Intelligence and Rare Disease Diagnosis (ARDD) is a recently developed AI-based framework for rare disease diagnosis. It processes multimodal medical data—including imaging, genomics, and EHR—to detect atypical symptoms and disease patterns, reducing misdiagnosis and missed cases (47).

Through breast imaging database establishment and deep learning (DL) optimization, AI technology can be assisted in breast cancer diagnosis (48). Through advanced image pattern recognition, AI algorithms can analyze medical imagery to accurately diagnose conditions such as extracervical resorption (ECR) of teeth (49), cataracts and retinopathy (50), and malignant and precancerous skin lesions by identifying disease-specific visual features imperceptible to the human eye (51). By analyzing long-term trends in patient behavior and symptoms, chatbots—which are AI-driven tools—help diagnose mental health disorders and enable the early detection of problems like anxiety and depression (52). Aptamer-functionalized field-effect transistor (FET) biosensors are used for detection of biomarkers related to tumors, nervous system and cardiovascular diseases for disease diagnosis (53).

Clinical diagnostics also makes extensive use of machine learning (ML), utilizing algorithms to identify patterns in data (54). Utilizing a supervised learning pipeline model for deep echocardiography, researchers first extract pertinent cardiac structural features ahead of training a semi-supervised generative adversarial network (GAN) model end-to-end with a convolutional neural network (CNN) classifier in the field of automated heart disease diagnosis (55). DL, a subset of ML, has witnessed a substantial rise in its applications within the field of dentistry over the last decade (56). For example, dental DL was applied to the analysis of maxillofacial radiographs for the diagnosis of temporomandibular joint disease, osteonecrosis of the jaw, or oral squamous cell carcinoma (57). ML algorithms may specifically detect polyps in real-time during colonoscopies (58), and CNNs may effectively assist healthcare providers in assessing brain tumor symptoms to determine disease severity (59).

### 4.3 Clinical treatment

Digital health introduces innovative tools and methods in clinical treatment. Remote blood glucose monitoring combined with AI algorithms, generates virtual models of diabetes to optimize insulin dosage and lifestyle guidance (60), enabling patients to maintain a high quality of life while alleviating the burden on the healthcare system (61). Drug 3D printing analyzes biomedical data to design precise printing structures, enhancing drug solubility and ensuring controlled, accurate drug release (62). The WHO has identified antimicrobial resistance (AMR) as one of the greatest threats to global health, food security, and development (63). The Electronic Point-of-Care Tests (e-POCT), a new electronic clinical decision support algorithm (CDSA), assists healthcare providers in evaluating symptoms and signs, helping to reduce antibiotic use and mitigate AMR concerns in children (64). This tool is poised to become pivotal in managing infectious diseases in children globally, offering crucial technical support in AMR prevention and control.

Virtual reality (VR) technology presents innovative strategies for symptom management and treatment strategies in patients with rheumatic and musculoskeletal disorders (RMDs) (65). VR provides non-pharmacological treatments through immersive environments and interactive experiences, alleviating pain (66). In neurological conditions, immersive VR meditation (IVRM) has shown significant effectiveness in reducing depression and anxiety symptoms through emotion regulation (67). Cognitive behavioral therapy (CBT) and other evidence-based therapies improve patient access to care (68). VR combined with CBT (VRC-CBT) demonstrates positive effects on the motor, sensory, and action suppression functions of children with autism spectrum disorder (ASD) (69).

The integration of machine learning (ML) and AI significantly enhances the safety of spinal surgery by analyzing preoperative data, formulating personalized treatment plans, and optimizing surgical decisions (70). In nerve damage cases, electron microscopy-based studies of the connectome aid in reconstructing damaged neural circuits (71). Semi- or fully autonomous robots are expected to perform delicate intraocular surgeries in the future (72). Furthermore, 3D-printed complex bone scaffolds offer new possibilities for the personalized treatment of bone defects (73). In dental treatments, AI and ML algorithms automate the design of dental restorations, including oral scanning, grinding, and 3D printing (74). The deep learning (DL) radiation therapy segmentation algorithm used for image reconstruction achieves expert-level accuracy, enhancing the efficiency of radiation therapy (75). Future research will focus on exploring and validating the clinical applications of additional DHTs.

### 4.4 Clinical prognosis

Digital health is transforming clinical prognosis. Wearable devices are increasingly being integrated into healthcare to continuously track and monitor patient indicators and manage the prognosis of cardiovascular disease (76), epilepsy (33), chronic diseases such as diabetes (41), hypertension (77), etc. Telemedicine platforms can ensure treatment compliance for tuberculosis patients (78), manage chronic diseases such as hypertension and reduce hospitalizations through regular remote follow-up (79), and digital platforms can assess the effectiveness of breast cancer healthcare resource utilization (80) and improve information accessibility and self-efficacy of oncology patients (81). ML is also used in the field of psychiatry to predict the onset of schizophrenia and the effectiveness of antidepressant drugs (82). Proprioceptive visual feedback may be a useful way to enhance motor control during rehabilitation training. Rheumatic and musculoskeletal diseases (RMDs) (65) and stroke patients significantly improved motor control (83) and balance after VR proprioceptive feedback training (84).

With the integration of digital health in healthcare, many prognostic models have merged. For patients with cancer, incorporating important biomarkers into clinical staging systems may improve risk stratification (85). Specifically, leveraging immune-related long non-coding RNA (lncRNA) markers, artificial intelligence algorithms demonstrate significant potential for predicting clinical outcomes, thereby advancing tumor therapy and paving the way for improved management strategies in the era of precision medicine (86). Furthermore, the DL-based multimodal fusion (MMF) algorithm analyzes cancer deaths, interprets histopathological features and molecular signatures, and evaluates prognostic indicators across patients (87). Additionally, the placenta model is capable of accurately predicting the outcome of pharmacological treatment in along with pregnancy syndrome (88). Meanwhile, ML and DL models are also used in predicting long-term disease progression in patients with chronic obstructive pulmonary disease (COPD) (89). These models’ prediction accuracy remains to improve better, offering solid proof for clinical judgment.

### 4.5 Public health

The influence of digital health on global public health gets particular attention during the 2020 Riyadh Global Digital Health Summit. To obtain the optimal global response, it is advised that nations enhance their digital health infrastructure, develop technical skills in digital health, adopt the global minimum data set, and adapt data treatment structures for infectious diseases (90). A branch of digital health called m-health supports public health initiatives (91) and has enormous promise for expanding access to healthcare, particularly in rural and underdeveloped areas (92). Another branch of digital health, RPM leverages technology to facilitate monitoring and shift disease management to the home setting, with patient satisfaction regarding comfort, equipment, communication, and overall experience reaching 88.97% (93).

For countries prone to natural disasters, the implementation of e-Nabiz applications can effectively alleviate severe disruptions to health care services caused by disasters (94). According to some academics, this project ranks among the most potent healthcare infrastructures. Furthermore, the utilization of drones in disaster response extends beyond the delivery of medical supplies, first assistance, blood sample transportation, and even emergency evacuation (95). Individuals with the means and availability to utilize digital mental health resources are increasingly transitioning from traditional in-person support services to online mental health platforms (96).

With the help of apps, AI and ML, public health agencies can enhance disease surveillance. Digital health offered a platform for monitoring the COVID-19 pandemic, and machine learning (ML) will be used to examine the combination of nucleic acid data from multiple sources, allowing for the early detection and prediction of future disease outbreaks (97). Meanwhile, AI and DL enhanced the detection and diagnosis of the virus and quickly analyzed the results (98). The use of biosensors may allow non-professionals to conduct professional accounting detection and real-time monitoring, increase detection speed, and reduce costs (99), reducing the spread of the virus and saving many lives (23). The comprehensive application of DHTs to make public health management more personalized will pave the way for a more efficient and equitable healthcare system, so we need to put these digital health tools into more practice (24).

### 4.6 Medical education and scientific research

Enhancing future physicians’ digital health competencies is crucial for improving healthcare quality. Developing targeted courses can equip medical students with the clinical skills needed to effectively leverage DHTs (100). A University of Queensland survey revealed strong student interest in digital health, with respondents emphasizing its relevance to future medical practice and advocating for its integration into core curricula (101). For instance, Semmelweis University’s popular elective course successfully enhances digital literacy, preparing students for the pervasive use of internet-based technologies in medicine (102).

As hubs of healthcare, research, and education, Academic Medical Centers (AMCs) must prioritize digital health training to advance both clinical care and scientific innovation (103). AMCs are pivotal in driving large-scale digital health initiatives, such as design labs, whose success depends on strategically coordinated advancements (104, 105). Many DHTs, such as head-mounted magnetic equipment and holographic 3D-printed models, are used in medical education for repeated practice (106). Progressive dental image generative adversarial network (PGGAN) generates arbitrary images that do not contain private information, data for DL and dental education (107). The rapid integration of DHTs is fundamentally transforming traditional medical education and research paradigms.

## 5 Discussion

DHTs have demonstrated transformative potential across healthcare, spanning disease prevention, diagnosis, treatment, and prognosis, thereby significantly enhancing the efficiency, quality, and safety of medical services. The integration of AI, wearable devices, and remote monitoring systems has not only refined clinical decision-making but also improved patient experiences and healthcare accessibility. Nevertheless, despite these advancements, the widespread adoption of digital health faces persistent challenges, including the digital divides, technical and regulatory constraints, privacy and data security concerns, and barriers to clinical workflow integration. Unresolved, these issues may impede the further evolution and practical efficacy of DHTs. The following discussion critically examines these challenges and proposes actionable solutions to inform policymakers and stakeholders, fostering sustainable integration and innovation in healthcare systems.

### 5.1 Digital health grapples with many challenges

#### 5.1.1 Digital ethics and equity

The widespread adoption of DHTs is significantly constrained by issues of digital ethics and equity, with the digital divide and algorithmic bias representing two central challenges. Significant disparities in digital literacy and access to communication technologies—across age, race, socioeconomic status, and geographic regions—not only constitute a digital divide (108) but also exacerbate health inequities and contribute to “health data poverty” (109) Without the necessary digital literacy, both patients and healthcare professionals will struggle to effectively utilize, interpret, and implement the data generated by DHTs. Limited digital literacy impedes effective use and interpretation of DHT-generated data by both patients and providers. Accessibility involves not only hardware but also inclusive design, multilingual support, and cultural adaptability. Income inequality and high research and development costs (110) further raise barriers to access, while a lack of robust evidence tailored to vulnerable populations (111) perpetuates the digital divide. This gap stems from uneven digital literacy, inequitable DHT access, and biased cost–benefit assessments of innovations—collectively hindering DHT dissemination.

Beyond these issues, DHTs raise broader ethical and equity concerns. Algorithmic bias in AI diagnostics disproportionately harm minority and low-income populations. A notable example is a cost-based algorithm that systematically underestimated the severity of black patients, resulting in a more than 50% reduction in their referrals for additional care (112). These risks are heightened in low- and middle-income countries (LMICs) due to unrepresentative data and weak local oversight. Even proven technologies are not adopted equitably. Less than 50% of patients with cardiac implantable defibrillator (ICD) have registered RPM systems, and RPM utilization is very low (113). If there are structural barriers to its promotion and use, it may in turn exacerbate the existing gap. The widespread use of DHT may have unintended ethical consequences: overreliance on automation risks eroding patient trust, while unregulated data use may worsen resource inequities. Excessive dependence on EHRs also limits alternative solutions and compromises care efficiency (114).

#### 5.1.2 Technical and regulatory policy restrictions

The accuracy of algorithms and the reliability of clinical decisions are compromised by the noise and inconsistent formatting of health data, rendering clinical big data vulnerable to potential threats related to validation and accuracy (115). Over the past decade, the application of DHTs in dentistry has expanded significantly; however, technologies such as chatbots remain unreliable for clinical decision-making in oral and maxillofacial surgery (OMFS) (56). The lack of flexibility in data retrieval, collection, processing, and storage methods remains a significant obstacle to the effective use of digital big data (116). Recently, the term “biotech syndrome” has been used to describe the health risks arising from the intersection of digital technologies and human physiology. Malfunctioning digital implants, such as insulin pumps or pacemakers, pose severe threats to patient health if they fail or malfunction (117).

A critical and complex legal challenge is determining accountability for errors or adverse effects resulting from DHTs. While the General Data Protection Regulation (GDPR) outlines the responsibilities of data processors and controllers in cases of improper use or poor decision-making related to personal data, the allocation of duties remains unclear due to the complexity of AI (118). The progress of digital health development is hindered by outdated legislation, unresolved legal issues surrounding technology, and uncertainty faced by developers and healthcare providers. Furthermore, insufficient legislative incentives and challenges in medical oversight complicate the engagement of companies in the healthcare sector (25).

#### 5.1.3 Privacy and data security concerns

The ownership of data within digital health systems remains unresolved, encompassing user-researcher rights and cross-border data use and regulation. The WHO advocates for an ethical framework governing cross-border health data exchange, emphasizing the importance of data sovereignty to protect national control over medical data (15). As international collaboration in data analysis increases, data ownership and privacy protection become more complex. Re-identification risks, associated with sharing personal data with research institutions, pose significant dangers, potentially leading to data misuse and privacy breaches (119, 120).

These concerns not only erode patient trust in digital health systems but may also prevent physicians from accessing complete patient data, thereby compromising the accuracy of diagnoses and treatment plans. Furthermore, cybersecurity vulnerabilities continue to threaten data security. Healthcare breaches in the U. S. surged by 55.1% in 2020 compared to 2019 (121). The growing use of AI in healthcare has also raised ethical challenges across individuals, relationships, institutions, and societies, making it difficult for healthcare systems to move beyond traditional models and impeding improvements in patient safety, service quality, and operational efficiency.

#### 5.1.4 Clinical workflow integration issues

Clinical data sets specific criteria that DHTs should meet when collecting data, including completeness, relevance, availability, security, and reliability (122). Given the complexity and high stakes of healthcare systems, the adoption of new DHTs remains challenging. Integrating diverse medical technologies, data, and processes effectively into clinical practice to improve healthcare quality and safety remains a formidable task. Interoperability issues arise when different medical systems—such as EHRs, imaging systems, and laboratory systems—use incompatible technical standards and data formats. Between 2007 and 2018, improper EHR deployment and interoperability issues led to 18,000 EHR-related patient safety incidents in the United States (123).

Before DHTs can be widely applied, sufficient evidence must be gathered to assess their impact on healthcare (25). Currently, a significant amount of medical data lacks interoperability, complicating data processing, interpretation, and exchange (14). A misalignment between clinical needs and technological solutions often leads to resource wastage and prevents DHTs from reaching their full potential. Additionally, the increasing complexity of operating systems has burdened clinical staff, reducing overall work efficiency.

The multifaceted challenges spanning technological constraints, policy deficiencies, and implementation difficulties highlight the critical need for comprehensive strategies to bridge the gap between technological innovation and practical application. The following discussion will systematically examine targeted solutions to address these barriers, providing stakeholders with pragmatic implementation pathways. These approaches are designed to ensure equitable access, maintain robust security, and achieve seamless clinical integration, thereby maximizing the transformative potential of digital health technologies.

### 5.2 Stakeholder-specific mitigation strategies for addressing digital health barriers to improve quality and safety of healthcare

#### 5.2.1 Prioritizing digital ethics and equity in development

Adressing ethics and equity issues is a prerequisite for safeguarding healthcare access and safety. It ensures that all patient populations can benefit from digital health technologies safely and effectively, thereby preventing the exacerbation of existing health disparities or the introduction of new medical risks due to inadequate access or design flaws. This necessitates policy-driven approaches that are operationalized and contextualized for diverse healthcare systems.

Policymakers should enhance and fund health systems by mandating interoperability standards, such as Health Level 7 Fast Healthcare Interoperability Resources (HL7 FHIR, in public procurement to enable seamless integration of technologies (124), developing a continuous ethical assessment mechanism and fairness assessment framework for different populations to ensure ethical standards, while implementing financial incentives—such as reimbursement codes for telehealth and RPM—to encourage adoption (125). In LMICs, priority should be given to scalable, low-cost investments, including public-private partnerships to expand broadband access in underserved and rural communities, which is a prerequisite for most DHTs. Concurrently, efforts to improve digital literacy through community engagement and integrate digital health interventions (DHI) into systemic planning are critical (110). As demonstrated in 2022 by the U. S. Department of Health and Human Services’ allocation of over $55 million to Health Resources and Services Administration (HRSA)-funded health centers to promote telehealth and address digital disparity issues (126), addressing digital disparities and literacy in the evolving tech landscape.

Technology developers must enhance accessibility through inclusive design principles. This includes implementing low-bandwidth optimization to ensure stable functionality of applications and platforms in environments with intermittent or slow internet connectivity (127)—a common scenario in LMICs and rural regions worldwide. Furthermore, accessibility by design should be prioritized, integrating aging-friendly interfaces, multi-sensory interactions (128), lightweight technologies, AI, and humanized interactions [e.g., Mind-Mate (129)]. Content must be culturally adapted, with health information, imagery, and guidance tailored to diverse cultural contexts and social norms to improve engagement and effectiveness.

To address digital literacy gaps, a multi-stakeholder approach is essential. Healthcare providers and implementers should incorporate digital health competency training into continuing medical education, conducting regular ethical and equity assessments of DHTs to promptly identify and rectify biases. Public health initiatives, led by community health workers, can establish digital navigation services, through which trusted personnel assist patients in setting up and using basic digital health tools—such as RPM devices or patient portal applications. For instance, electronic Patient-Reported Outcome (ePRO) systems designed for complex chronic disease management enable collaborative goal-setting between patients and providers via mobile-linked portals, facilitating progress tracking between appointments (130).

#### 5.2.2 Breaking technical and policy restrictions will drive the accelerated development of digital health

To enhance the reliability and safety of DHTs, stakeholders must address technical limitations and regulatory ambiguities through evidence-based validation, interoperable design, and supportive policy frameworks. Meta-analysis of molecular big data can be used to increase statistical accuracy and improve the credibility of research findings (116). Validating the clinical significance and reproducibility of results is a critical research concern (131). Medical providers and implementers should validate the clinical significance of digital health technologies (DHTs) through rigorous real-world testing, peer-reviewed outcome studies, and small-scale pilot programs within existing regulatory frameworks to gather evidence, ensure efficacy and safety, and iteratively adapt solutions before scaling deployment.

To mitigate risks for developers and encourage adoption, clearer legal frameworks and guidelines on usage, monitoring, and accountability are necessary. Policymakers should further promote innovation by offering incentives such as tax breaks and subsidies (1), thus fostering private sector investment and enabling the creation of accessible and affordable digital health solutions for underserved communities. Patients and the public should engage in policy discussions, support inclusive design initiatives, and advocate for equitable access to advance digital health tools that meet real-world needs. They can also join patient organizations to amplify collective advocacy and monitor updates from international agencies and local policymakers to promote technology fairness, privacy protection, and accessibility.

#### 5.2.3 Privacy and data security are prerequisites for digital health development

Unlocking the potential of de-identified health data while safeguarding patient confidentiality and trust requires policymakers to establish comprehensive regulations that integrate technical, legal, and ethical frameworks in order to protect health data security and personal privacy (132). For instance, the GDPR mandates that patient data be collected and utilized in a lawful and equitable manner, establishing guidelines to ensure the confidentiality of personal data (118).

Simultaneously, technology developers should invest more heavily in privacy protection technologies. By promoting the creation of solutions that effectively prevent the identification of personal data, while embedding privacy and security principles into the design process of digital health products. Federated learning, a novel AI approach, offers decentralized algorithm training to address privacy and data security concerns without requiring data sharing (133). The de-identification and anonymization of data will facilitate the reuse of otherwise sensitive or legally restricted information, supporting the efficacy and scalability of digital health initiatives (134).

Reducing the burden on healthcare providers and implementers in managing data security responsibilities is also crucial. It is recommended that responsibilities be clearly delineated in collaboration with technical departments to ensure accountability. Furthermore, broad public education initiatives that inform patients and the general public about how their health information is used will empower individuals to make informed decisions based on their personal circumstances. Such initiatives will also help build trust in DHTs (135) and provide a strong social foundation for addressing the challenges of integrating these technologies into sustainable clinical workflows.

#### 5.2.4 Effective digital health hinges on clinical workflow integration

Collaboration among multiple stakeholders is essential for the successful integration of clinical activities. Policymakers should establish a utility-centered evaluation framework to conduct rigorous real-world evidence (RWE) assessments for DHTs, with key metrics focusing on clinical utility and workflow integration rather than solely on technical performance. Clear integration guidelines should be issued to define clinical workflow standards that developers must meet, thereby streamlining the regulatory and approval processes for digital health products. Additionally, the healthcare payment system should be reformed by creating new billing codes specifically to reimburse services that are delivered through certified DHTs and integrated into clinical workflows.

To ensure the efficient deployment of medical resources and a comprehensive improvement in service quality, technology developers must create secure, reliable platforms for data sharing, optimize clinical workflows through process reengineering and standard operating procedures, establish interdisciplinary collaboration mechanisms, and develop scientific evaluation systems. Technicians may consider developing AI clinical frameworks similar to Artificial Intelligence and Rare Disease Diagnosis (ARDD), which could provide structured diagnostic interfaces, integrate multi-dimensional patient data, synthesize analytical results from various modules, and generate preliminary diagnostic suggestions to assist clinicians in the treatment process (47).

By combining FHIR-based interoperability with blockchain-based data provenance and security mechanisms, healthcare organizations can establish a robust technical foundation for trustworthy and efficient health information exchange to ensure healthcare quality and safety. Continuous data feedback should inform dynamic adjustments to integration plans. Middleware technology (136) and standardized protocols like HL7 FHIR (124) can facilitate seamless data sharing across diverse medical systems through defined interfaces for core resources such as Observation, DiagnosticReport, and MedicationRequest. In parallel, blockchain technology offers a novel, decentralized solution to link EHRs and address common clinical challenges related to data integrity, auditability, and patient consent management (137).

Change management, progressive implementation, and increased medical acceptability are critical components for healthcare providers. For successful digital health integration, patients and the public should participate in third-party certification processes. Clinical process integration can be achieved through methodical approaches, multi-party collaboration, and continuous development. Successful integration will optimize resource allocation, significantly enhance healthcare quality and safety, and ultimately provide patients with better and more efficient medical care.

Finally, by evaluating multiple digital health technology assessment (dHTA) in the implementation of digital health—such as population coverage rate of digital health population, regulatory approval rate, algorithm accuracy and transparency, clinical efficiency, and rate of data security breaches, etc—we can determine whether the barriers faced by different stakeholders have been effectively addressed (138) (Table 1).

**Table 1:** Stakeholder-specific mitigation strategies for addressing digital health barriers.

Barriers	Mitigation strategies of different stakeholders				Metrics	References
	Policymakers	Technology developers	Medical providers and implementers	Patients and public		
Digital ethics and equity	Mandate interoperability standards (e.g., HL7 FHIR; Integrate digital health interventions (DHI); Develop an ethical and fair evaluation framework.	Implement low-bandwidth optimization; Enhance technological accessibility Design cultural adaptation.	Incorporate digital health competency training into continuing medical education; Conduct regular ethical and equity assessments.	1. Establish digital navigation services; 2. Engage public education on DHTs.	1. Coverage rates of digital health population; 2. Digital literacy adaptability rates; 3. Algorithm accuracy and transparency rates; 4. Health benefits rates, etc.	(110, 124–130, 138)
Technical and regulatory policy restrictions	1. Expand legislative incentives (e.g., tax breaks and subsidies); 2. Develop legal frameworks.	1. Enhance databases; 2. Ensure interoperability and data credibility; 3. Expand research.	1. Conduct rigorous real-world testing; 2. Find peer-reviewed outcome studies; 3. Launch small-scale pilot programs.	1. Engage in policy discussions; 2. Support inclusive design initiatives; 3. Advocate for equitable access; 4. Join patient organizations and monitor updates from international agencies and local policymakers.	1. Technical credibility; 2. Regulatory approval rate; 3. Standard interoperability protocol adoption rate, etc.	(1, 116, 131, 138)
Privacy and data security concerns	1. Establish data protection regulations; 2. Promote ethical frameworks.	1. Develop privacy protection technology (e.g., de-identification and anonymization; 2. Implement federated learning.	1. Embed joint review with technical departments; 2. Manage data security.	1. Public education campaigns; 2. Navigate personalized decisions on data sharing.	1. Rate of data security breaches; 2. Privacy design principles embedding; 3. Data localization and cross-border transmission compliance, etc.	(118, 132–135, 138)
Clinical workflow integration issues	1. Establish a utility-centered evaluation framework; 2. Reform health insurance payment policies and create new billing codes; 3. Clear integration guidelines.	1. Optimize clinical workflows; 2. Develop data sharing platform and standardized protocols (e.g., middleware and blockchain).	1. Change management; 2. Adopt progressive implementation; 3. Obtain third-party certification	1. Participate in certification; 2. Engage in multi-party collaboration	1. Clinical efficiency (e.g., readmission rate within 30 days); 2. Patient and healthcare satisfaction, etc.	(47, 124, 137, 138)

HL7 FHIR, Health Level 7 Fast Healthcare Interoperability Resources; DHI, digital health interventions; DHTs, digital health technologies.

## 6 Outlook

Building upon the challenges and solutions discussed, the future development of digital health technologies requires coordinated efforts across multiple dimensions. First, establishing standardized validation protocols will be critical to ensure the reliability and clinical applicability of emerging technologies. Second, policy interventions must be implemented to address existing disparities in digital access and literacy, particularly for vulnerable populations. Third, the development of robust regulatory frameworks should balance innovation with patient safety, creating an environment conducive to responsible technological advancement.

Looking ahead, three priority areas demand particular attention: (1) the creation of evidence-based implementation frameworks to facilitate DHT integration into clinical workflows, (2) advancement of federated learning approaches to enhance data security while enabling collaborative research, and (3) formulation of international standards for technology evaluation and governance. The successful realization of digital health’s full potential will necessitate unprecedented collaboration among healthcare providers, patients, policymakers, and technology developers, with a shared commitment to improving healthcare quality and safety worldwide.

## 7 Conclusion

This review substantiates DHTs as transformative elements in modern healthcare, demonstrating significant improvements in diagnostic accuracy, treatment efficacy, and healthcare accessibility. While AI, wearables, and telemedicine have enhanced patient outcomes and resource efficiency globally, the COVID-19 pandemic both validated their utility and exposed systemic vulnerabilities in digital infrastructure and equity. As healthcare systems worldwide confront growing demands and evolving challenges, DHTs represent not merely adjunct tools but fundamental components of sustainable, patient-centered care models. Their continued evolution and responsible implementation promise to redefine healthcare quality and safety standards in the coming decades, ultimately fulfilling the vision of precision medicine accessible to global populations.

## Glossary

WHO: World Health Organization
COVID-19: Coronavirus Disease 2019
DHTs: digital health technologies
IoT: Internet of Things
telemedicine: telecommunications medicine
IT: information technology
EHRs: electronic health records
PGHD: patient-generated health data
DTx: Digital therapeutics
RPM: remote patient monitoring
HF: heart failure
AI: artificial intelligence
POMR: Problem-Oriented Medical Record
COSTAR: Computer Stored Ambulatory Record
HELP: Health Evaluation through Logical Processes
e-health: electronic health
m-health: mobile health
GIDH: Global Initiative for Digital Health
CBT: cognitive behavioral therapy
AF: atrial fibrillation
DeepMPTB: Deep Learning Model for Preterm Birth Prediction
CGM: continuous glucose monitoring
ARDD: Artificial Intelligence and Rare Disease Diagnosis
DL: deep learning
ECR: extracervical resorption
FET: field-effect transistor
ML: machine learning
GAN model: generative adversarial network model
CNN: convolutional neural network
AMR: antimicrobial resistance
CDSA: clinical decision support algorithm
VR: virtual reality
RMDs: rheumatic and musculoskeletal disorders
MMF: multimodal fusion
IVRM: immersive VR meditation
CBT: cognitive behavioral therapy
RC-CBT: VR combined with CBT
ASD: autism spectrum disorder
RMDs: rheumatic and musculoskeletal diseases
COPD: chronic obstructive pulmonary disease
AMC: Academic Medical Centers
PGGAN: progressive dental image generative adversarial network
LMICs: low- and middle-income countries
ICD: Implantable cardioverter-defibrillator
DHI: digital health interventions
OMFS: oral and maxillofacial surgery
GDPR: General Data Protection Regulation
HL7 FHIR: Health Level 7 Fast Healthcare Interoperability Resources
HRSA: Health Resources and Services Administration
ePRO: electronic Patient-Reported Outcome
RWE: real-world evidence
ARDD: Artificial Intelligence and Rare Disease Diagnosis
dHTA: digital health technology assessment
