# Supplementary material for: Digital health: current applications, challenges, and future directions for enhancing healthcare quality and safety
Source: Front Public Health. 2025 Sep 26;13:1646802. doi: 10.3389/fpubh.2025.1646802 (PMC12516163; doi:10.3389/fpubh.2025.1646802)
Supplement: Supplementary file 1 [file Data_Sheet_1.pdf]

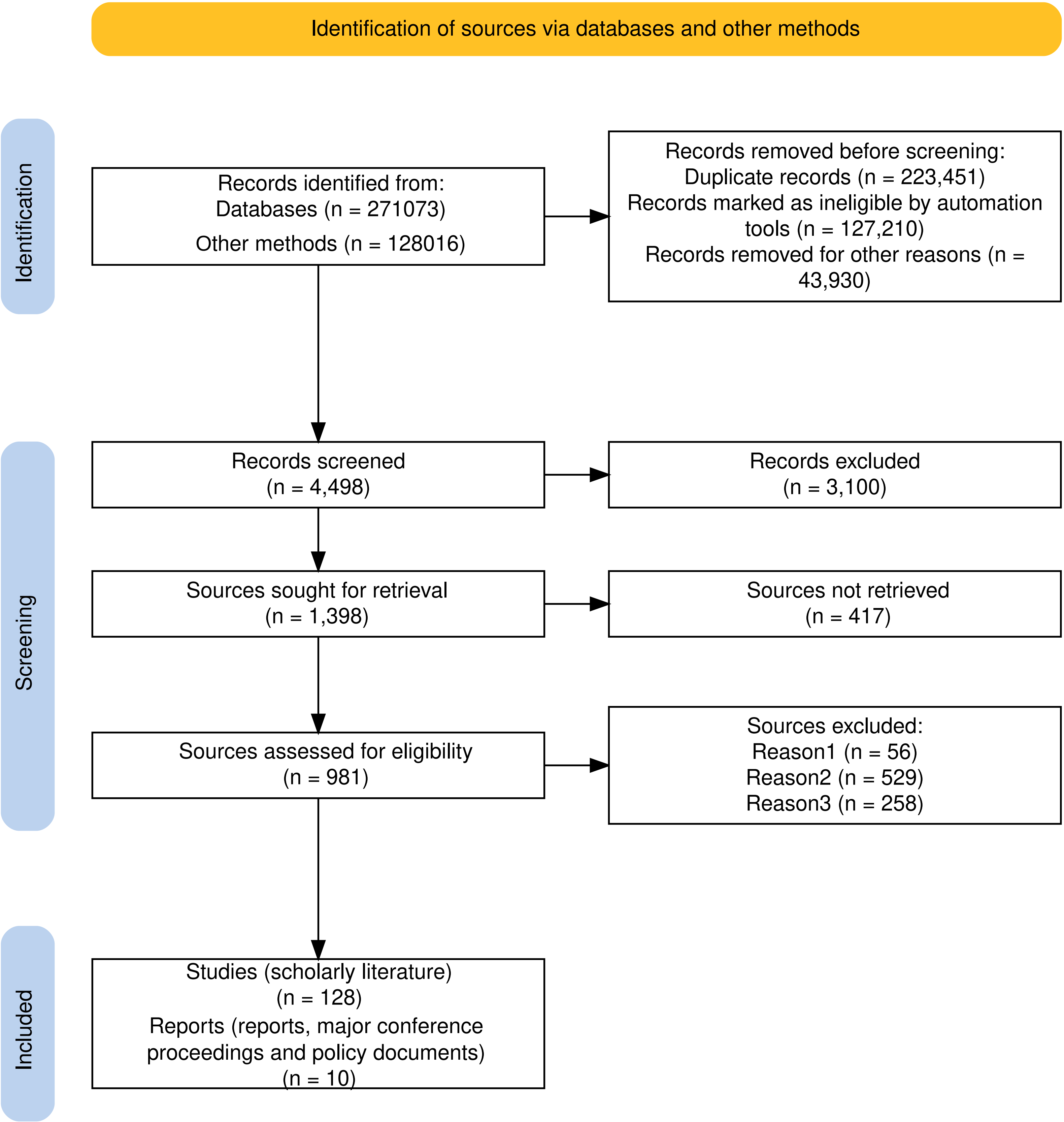
Identification of sources via databases and other methods
Identification
Screening
Included
Records identified from:
Databases (n = 271073)
Other methods (n = 128016)
Records removed before screening:
Duplicate records (n = 223,451)
Records marked as ineligible by automation tools (n = 127,210)
Records removed for other reasons (n = 43,930)
Records screened
(n = 4,498)
Records excluded
(n = 3,100)
Sources sought for retrieval
(n = 1,398)
Sources not retrieved
(n = 417)
Sources assessed for eligibility
(n = 981)
Sources excluded:
Reason1 (n = 56)
Reason2 (n = 529)
Reason3 (n = 258)
Studies (scholarly literature)
(n = 128)
Reports (reports, major conference proceedings and policy documents)
(n = 10)
